# Anorexia Nervosa, Anxiety, and the Clinical Implications of Rapid Refeeding

**DOI:** 10.3389/fpsyg.2018.01097

**Published:** 2018-07-04

**Authors:** Sarah Kezelman, Ross D. Crosby, Paul Rhodes, Caroline Hunt, Gail Anderson, Simon Clarke, Stephen Touyz

**Affiliations:** ^1^School of Psychology, University of Sydney, Sydney, NSW, Australia; ^2^Neuropsychiatric Research Institute, Fargo, ND, United States; ^3^School of Medicine & Health Sciences, University of North Dakota, Fargo, ND, United States; ^4^Department of Adolescent and Young Adult Medicine, Westmead Hospital, Sydney, NSW, Australia

**Keywords:** anorexia nervosa, adolescent, refeeding, weight gain, eating disorders

## Abstract

The current study aimed to examine the temporal relationship between anxiety symptoms and weight gain for adolescents with anorexia nervosa over the course of an inpatient admission targeting weight restoration through rapid refeeding. Participants were 31 females presenting to a specialist inpatient unit. Psychometric assessments using standardized procedures were conducted to assess co-morbid anxiety diagnoses, and eating disorder symptom severity at admission and discharge. Study protocols were completed on a weekly basis over the course of their admission and were compared with weekly BMI change. Multiple mixed-effects linear models with random intercepts were used to assess change in weight status and psychological variables. Results indicated a reduction in anxiety over the course of hospitalization; however, there was no evidence to support a relationship between anxiety change and weight restoration. The clinical implications of these results are discussed and directions for future research recommended.

## Introduction

Inpatient admissions aimed at correcting the biological sequelae associated with severe malnutrition and self-starvation are recommended as a principal treatment intervention for anorexia nervosa (AN; American Psychiatric Association [APA], 2013). Despite the importance of nutritional rehabilitation, no definitive recommendations for refeeding protocol in an inpatient setting exist. Clinical practice guidelines (National Collaborating Centre for Mental Health [NICE], 2004; American Psychiatric Association [APA], 2006; The Society for Adolescent Health Medicine [SAHM], 2015) tend to provide a comprehensive overview of widely varied approaches to refeeding, necessarily referring back to a reliance on clinical expertise and discretion due to the paucity of rigorous empirical literature. Unsurprisingly, this marked variability is reflected in clinical practice with disparate approaches evident in regard to refeeding method [e.g., traditional bolus feeds vs. nasogastric (NG) feeding], total daily caloric intake, length of hospitalization, and weighing protocol (Maguire et al., 2003; Schwartz et al., 2008).

Traditionally, cautious approaches to refeeding have been promoted. This is largely due to the potential dangers associated with refeeding syndrome, which describes the metabolic and clinical disturbances that can occur, most commonly in the first week of starting to refeed patients with AN. These can include abnormalities of glucose metabolism, as well as altered serum electrolytes (specifically lower levels of phosphate, potassium, and magnesium), thiamine deficiency, and sodium and fluid retention. If untreated, these disturbances can be life threatening and lead to a number of complications including delirium and seizures, organ failure, cardiac arrhythmias, and sudden death (Kohn et al., 2011; Katzman et al., 2014). Close monitoring and timely supplementation of serum electrolytes prevents the development of this potentially life threatening syndrome.

Emerging literature espouses that the caloric prescriptions previously recommended (e.g., initiating patients on a daily caloric intake of approximately 1,200 kcal/day and advancing diets by 200 kcal/day; National Collaborating Centre for Mental Health [NICE], 2004; American Psychiatric Association [APA], 2006), may be insufficient to achieve recommended rates of weight gain required for AN patients presenting to an inpatient unit (Wandler, 2012; Hay et al., 2014). It has also been suggested that rigorous medical monitoring and the implementation of adjunctive nutritional supplementation can effectively control for physiological risks (e.g., refeeding syndrome) associated with refeeding (Kohn et al., 2011). Consequently, research is increasingly focused on assessing the applicability and acceptability of more aggressive and rapid refeeding protocol.

To date, research findings support the medical safety of rapid NG refeeding. Efficient weight gain can be achieved by initiating patients on higher caloric diets and rapidly advancing total daily caloric intake; without consequential increases in episodes of refeeding syndrome and/or increased risks related to severe electrolyte abnormalities (Whitelaw et al., 2010; Agostino et al., 2013; Garber et al., 2013; Golden et al., 2013; Leclerc et al., 2013; Redgrave et al., 2015). Significant associated reductions in the duration of hospitalization (Agostino et al., 2013; Garber et al., 2013; Golden et al., 2013) and conceivable concomitant financial and psychosocial benefits have further promoted the potential utility of these approaches. Some research has raised concerns regarding the potential psychologically aversive effects of such aggressive protocols for patients with AN (Vandereycken, 2003), though none of the aforementioned studies included an investigation of psychological factors.

In fact, research assessing the psychological acceptability and/or tolerability of rapid refeeding approaches is extremely limited. This dearth of research seems enigmatic in a population diagnostically defined by the presence of an intense fear and/or behavioral interference with gaining weight (American Psychiatric Association [APA], 2013), core features specifically targeted in inpatient weight restoration. In fact, some authors have cautioned that discrepancies in the relative speed of physical and psychological recovery may hinder clinical progress and attainment of desirable outcomes (Fennig et al., 2002), a discrepancy that is conceivably amplified by the regular implementation of rapid refeeding protocol.

While it has been accepted that psychological features associated with AN are affected by the acute stages of malnutrition, questions remain as to whether these features can be regarded solely as complications of malnutrition or whether they should also be considered as unique psychiatric features independent of core AN symptomology. There is some research to demonstrate significant improvements in depressive symptoms following weight restoration (Mattar et al., 2011). This research has predominantly been conducted on a retrospective basis and for individuals with less severe and complex presentations of AN (Schlegl et al., 2014). Conversely, a growing body of literature asserts the persistence of psychological symptoms despite weight normalization (Bachner-Melman et al., 2006; Miller et al., 2009), with suggestions that changes in weight restoration alone are insufficient for the resolution of core psychological difficulties and achievement of long-term recovery (Dancyger et al., 2013). To clarify the abovementioned suggestions, a consideration of the temporal prospective relationship between physiological and psychological processes during the acute stages of weight restoration is needed.

A consideration of experiences of anxiety for individuals with AN has been highlighted in recent years as providing a meaningful avenue for informing conceptualizations of illness maintenance and consequently development of treatment interventions. This focus has been promoted due to the observed high rates of psychiatric comorbidity between AN and anxiety disorders (Swinbourne et al., 2012), neurobiological developments implicating a physiological overlap (Frank and Kaye, 2012), marked behavioral symptomological overlap (e.g., safety behaviors; Hildebrandt et al., 2012), and evidence stipulating the potential anxiolytic function of AN behaviors (Thornton et al., 2011). Furthermore, there has been some suggestion that the presence of anxiety symptomology in patients with AN may represent a negative prognostic factor (Zerwas et al., 2013). Thus, an assessment of anxiety processes may serve to delineate the abovementioned queries regarding the relationship between physical and psychological processes and further advance our understanding of individuals’ psychological experiences during acute nutritional rehabilitation of AN.

In addition, atypical anti-psychotic medications are increasingly used as an adjunctive treatment in the acute stages of refeeding in inpatient settings. This is despite the fact that no psychotropic medications are currently approved for the treatment of AN (Golden et al., 2013). Reasons implicated for the use of these psychopharmacological interventions include expected weight gain and/or an expected reduction in anxiety and agitation (McKnight and Park, 2010). Yet, research suggests that individuals with AN may be resistant to the typical weight gain enhancing side effects of antipsychotics and evidence regarding the psychological benefits of these medications is largely inconclusive (Hay and Claudino, 2002; McKnight and Park, 2010). Conceivably, this increased reliance on psychopharmacological intervention reflects an acute need to manage significantly high levels of clinical distress and anxiety observed during inpatient admissions, further contributing to the urgent need for empirical research to examine experiences of anxiety for an individual undergoing acute rapid nutritional rehabilitation.

Accordingly, the current study sought to address these research gaps. We aimed to further our understanding of anxiety symptoms and other related psychological experiences during acute stages of nutritional rehabilitation. Specifically, we aimed to clarify the temporal relationship between anxiety symptoms and weight change over the course of an inpatient admission while targeting weight restoration through rapid refeeding. While we expected evidence of psychological distress and anxiety over the course of hospitalization, given the dearth of current research, specific hypotheses regarding relational direction and/or influence were withheld.

## Materials and Methods

### Participants

Female adolescent patients hospitalized at a specialist inpatient medical unit for nutritional rehabilitation were enrolled in the current study between August 2013 and November 2014. Patients were ineligible to participate if they did not meet full DSM-5 (American Psychiatric Association [APA], 2013) diagnostic criteria for AN and/or had a comorbid diagnosis of psychosis, or intellectual disability. No other restrictions were placed on eligibility. Thirty-two eligible inpatients initially provided consent, representing approximately half of those approached; one patient later withdrew consent. As such, 31 participants aged between 15 and 19 years, with a mean age at admission of 16.91 years (*SD* = 1.10) were included in the study. Mean duration of hospital stay was 24.81 days (*SD* = 12.51, *Range* = 8–66) and approximately two-thirds of participants met criteria for a comorbid anxiety diagnosis. Two-thirds of participants in the current study were prescribed anti-psychotic medication, predominantly olanzapine, while in hospital. **Table 1** presents participant characteristics for the full sample.

**Table 1:** Participant characteristics for total sample (*N* = 31).

Characteristics	Mean	*SD*
Admission BMI (kg/m^2^)	16.31	1.94
Illness duration (months)	16.73	21.12
	
	***N***	**%**
	
AN subtype restricting	24	77.4
Binge-eating/purging	7	22.6
Previous admissions	6	19.35
Medication use prior to adm	6	19.35
Medication use during adm	20	64.52
Hx of DSH or suicidality	10	32.26
Any anxiety disorder diagnosis	20	64.52
Social phobia	12	38.71
Generalized anxiety disorder	14	45.16
Obsessive–compulsive disorder	2	6.45
Specific phobia	1	3.22
Agoraphobia	1	3.22

*BMI, body mass index; AN, anorexia nervosa; adm, admission; Hx, history; DSH, deliberate self-harm.*

### Procedure

Consecutive patients were approached and baseline measurements were collected within 72 h of admission. Diagnostic interviews for comorbid anxiety disorders were conducted within 1 week of admission. Participants completed self-report measures on a weekly basis during their inpatient admission, and final assessments were conducted within 48 h of discharge. Interviews were conducted by the primary researcher (SK), a clinical psychologist trained in diagnostic assessment. Demographic and weight data were collected from patient medical records. The relevant ethical review boards approved study protocol, and all participants provided informed consent. All parents were also informed, and for all participants under the age of 16 years, parental consent was obtained.

### Treatment Setting

This study was conducted at the Adolescent Medical Unit at Westmead Hospital, Sydney, NSW, Australia. Medically unstable patients are admitted to this unit for nutritional rehabilitation in order to treat and prevent further potentially severe physiological consequences associated with malnutrition. Rapid refeeding is achieved via concurrent use of NG tube feeding and standard oral feeds (daily calories divided into three meals and three snacks), thereby promoting a rapid advancement of caloric intake.

To avoid low blood sugar levels from the effects of delayed insulin phase and metabolic changes, patients are initiated at admission on continuous NG feeds at 2,400 kcal/day for a 24-h period. Patients are closely monitored and are also started on phosphate and multivitamin supplementation. Once medical stability is achieved, NG feeds are administered overnight only, with caloric intake provided via this method reduced as oral feeding increases. Each patient then progresses through a five-staged meal plan initiated at 1800 kcal/day with a maximal oral caloric intake of 3800 kcal/day. Specific daily caloric intake is individualized within these parameters according to patient medical need, reviewed thrice weekly, and trained nursing staff supervises all meals. All patients are referred on to outpatient psychological services prior to discharge. Specific discharge criteria can vary depending on the model of psychological follow-up; in general, patients must be medically stable, eating food (without the need for supplemental feeding), and have achieved a minimum healthy body weight (i.e., BMI ≥ 18.5).

Additional program components include ongoing engagement with school programming, thrice-weekly group therapy sessions led by an Occupational Therapist, a group led by a Clinical Psychologist, and twice weekly physiotherapy. The multidisciplinary team conducts weekly ward rounds and family interviews are held throughout the course of the admission to provide psycho-education and ensure appropriate outpatient follow-ups are coordinated. Minimal individual psychological intervention provided by part-time clinical psychologist is available on referral from the multidisciplinary team.

### Measures

#### Anxiety Disorder Interview Schedule, Child Version (ADIS-C; Silverman and Nelles, 1988)

The ADIS-C is a semi-structured clinical interview used to assess DSM-IV anxiety diagnoses and symptoms in children and adolescents. The ADIS-C has demonstrated good to excellent reliability across symptom scale scores (Silverman et al., 2001).

#### Eating Disorders Examination Questionnaire (EDE-Q; Fairburn and Beglin, 1994)

The EDE-Q is a 41-item self-report instrument adapted from the Eating Disorders Examination, which provides an overall global severity score and scores on four subscales of eating disordered behavior: restraint, shape concern, weight concern, and eating concern. The EDE-Q has excellent internal consistency and test–retest reliability (Luce and Crowthe, 1999).

#### Beck Anxiety Inventory (BAI; Beck and Steer, 1990)

The BAI is a 21-item self-report instrument designed to assess the severity of emotional, physiological, and cognitive symptoms of anxiety. Higher total scores reflect greater anxiety symptomology. The BAI has been found to have high internal consistency and moderate to high concurrent validity in an adolescent inpatient population (Jolly et al., 1993).

#### Speilberger State Trait Anxiety Inventory (STAI; Speilberger et al., 1970)

The STAI is a 40-item self-report instrument, with 20 items assessing for trait anxiety (STAI-Y1) and 20 items assessing for state anxiety (STAI-Y2). Total scores are calculated for each subscale, with higher scores indicating greater severity. The STAI has excellent internal consistency and good test–retest reliability.

#### Anxiety Control Questionnaire (ACQ; Rapee et al., 1996)

The ACQ is a 30-item self-report instrument designed to measure an individual’s perceived control over internal emotional anxiety-related experiences and reactions to anxiety-provoking external events. The ACQ has high internal consistency and has demonstrated good test–retest reliability.

#### Beck Depression Inventory (BDI-II; Beck et al., 1996)

The BDI-II is a 21-item self-report instrument that assesses DSM-IV diagnostic criteria for depression. Higher total scores demonstrate greater severity of depressive symptoms. The BDI-II has good psychometric properties.

### Statistical Analyses

Mixed-effects linear models with random intercepts were conducted using SPSS version 22.0 to evaluate changes in all anxiety and depression measures over time. Models included all available data and missing data were not imputed. BMI was added to the models as a time-varying covariate to evaluate the relationship between changes in weight and changes in anxiety or depressive symptoms. Additional secondary analyses were conducted by adding lifetime history of an anxiety disorder, admission BMI, duration of illness, or prescription of anti-psychotic medication during admission to the models as both a main effect and interaction with time to determine whether changes in anxiety or depressive symptoms over time differed as a function of these characteristics. Finally, paired samples *t*-tests were conducted to assess changes from admission to discharge in psychological variables, weight, and eating disorder symptom severity.

## Results

A series of mixed-effect model analyses were used to assess changes in dependent variables over time. Estimates revealed significant reductions in BAI (*p* < 0.05), STAI-Y1 (*p* < 0.05), and BDI (*p* < 0.001) scores over time. No significant changes in the STAI-Y2 or ACQ were found. Subsequent analyses adding BMI to each model as a time-varying covariate indicated that significant changes in BAI, STAI-YI, and BDI scores were not significantly associated with changes in BMI over time. **Table 2** provides further details of these analyses, and **Table 3** presents mean values at admission and discharge for these variables. Additionally, no main effect or interaction with time effect was found when lifetime history of an anxiety disorder, admission BMI, or duration of illness were added to the models. A significant interaction with time effect was found for BDI scores, *t* (96) = 2.24, *p* < 0.05, 95% CI [0.24, 4.00], when use of anti-psychotic medication was added to the model, such that individuals receiving medication experienced a greater reduction in depressive symptoms.

**Table 2:** Estimates for change in dependent variables over time and when BMI added as covariate.

Dependent variables	Independent variable	*t* (df)	Estimate	95% CI	*p* value
BAI	Time	-2.56	-1.00	-1.78, -0.22	0.012^∗^
	BMI	-0.73	-0.63	-2.35, 1.09	0.467
BDI	Time	-3.45 (98)	-1.62	-2.55, -0.69	0.001^∗∗^
	BMI	0.14 (72)	0.14	-1.91, 2.12	0.889
STAI-Y1	Time	-2.55 (102)	-1.43	-2.55, -0.32	0.012^∗^
	BMI	-0.40 (56)	-0.44	-2.63, 1.75	0.691
STAI-Y2	Time	-1.92 (96)	-0.81	-1.65, 0.03	0.058
	BMI	1.16 (77)	1.11	-0.79, 3.01	0.249
ACQe	Time	1.33 (96)	0.56	-0.27, 1.39	0.185
	BMI	-0.06 (53)	-0.05	-1.67, 1.57	0.953
ACQr	Time	1.47 (90)	0.56	-0.20, 1.32	0.145
	BMI	0.07 (83)	0.06	-1.83, 1.96	0.946

*BMI, body mass index; BAI, Beck Anxiety Inventory; BDI, Beck Depression Inventory; STAI-Y1, Speilberger State Trait Anxiety Inventory – Form 1; STAI-Y2,Speilberger State Trait Anxiety Inventory – Form 2; ACQe, Anxiety Control Questionnaire subscale for control over external events; ACQr, Anxiety Control Questionnaire subscale for control over internal reactions; ^∗^p < 0.05, ^∗∗^p < 0.01.*

**Table 3:** Paired samples *t*-test comparing admission and discharge scores on dependent variables.

	Admission		Discharge		Statistic
			
	*M*	*SD*	*M*	*SD*	*t* (df)
BAI	20.74	12.82	14.33	10.85	3.43 (26)^∗^
BDI	27.68	13.90	20.45	15.11	2.43 (21)^∗^
STAI-Y1	59.18	11.09	50.79	16.05	2.83 (27)^∗^
STAI-Y2	54.72	13.31	51.68	14.16	1.03 (24)
ACQe	43.00	9.80	45.73	12.54	-0.89 (21)
ACQr	33.00	11.10	34.36	14.66	-0.59 (24)

*BAI, Beck Anxiety Inventory; BDI, Beck Depression Inventory; STAI-Y1, Speilberger State Trait Anxiety Inventory – Form 1; STAI-Y2, Speilberger State Trait Anxiety Inventory – Form 2; ACQe, Anxiety Control Questionnaire subscale for control over external events; ACQr, Anxiety Control Questionnaire subscale for control over internal reactions; ^∗^p < 0.05.*

Paired samples *t*-tests were conducted to compare all anxiety and depression measures, EDE-Q global, subscale scores, and BMI at admission and discharge. There was a significant difference in scores for global EDE-Q (*p* < 0.05), Restraint subscale (*p* < 0.01), and the Eating Concern subscale (*p* < 0.05) over time, demonstrating a reduction in eating disorder severity on these subscales over time. No significant difference in scores for the Shape Concern or Weight Concern subscales was found. There was a significant difference in BMI from admission to discharge (*p* < 0.01), demonstrating an improvement in weight during admission. **Table 4** provides further information relating to these analyses.

**Table 4:** Change in EDE-Q and BMI score from admission to discharge.

	Admission		Discharge		Statistic
			
	*M*	*SD*	*M*	*SD*	*t* (df)
BMI (kg/m^2^)	16.32	1.94	19.36	1.15	-13.38 (30)^∗∗^
EDE-Q global	3.91	1.48	3.05	1.53	2.385 (21)^∗^
EDE-Q Restraint	3.86	1.70	1.59	1.71	5.45 (21)^∗∗^
EDE-Q Eating Concern	3.24	1.55	2.33	1.43	2.40 (20)^∗^
EDE-Q Shape Concern	4.32	1.46	4.14	1.83	0.44
EDE-Q Weight Concern	3.82	1.56	3.50	1.85	0.80

*M, mean; SD, standard deviation; BMI, body mass index; EDE-Q, Eating Disorders Examination Questionnaire. ^∗^p < 0.05; ^∗∗^p < 0.01.*

## Discussion

The present study utilized a prospective design to examine the temporal relationship between weight gain and anxiety over the course of an inpatient treatment for AN targeting nutritional rehabilitation through rapid refeeding. Results indicated that anxiety and depressive symptoms reduced over the course of hospitalization. Furthermore, significant improvements were observed in global eating disorder symptomology and subscales related to eating and restraint, consistent with previous literature (Madden et al., 2015). Thus, at first glance, and in line with literature exploring the medical safety and applicability of rapid refeeding protocols (Whitelaw et al., 2010; Agostino et al., 2013; Garber et al., 2013; Golden et al., 2013; Leclerc et al., 2013; Redgrave et al., 2015), these findings seem to provide some support for the permissibility of implementing such approaches.

There was, however, no evidence to support an association between the observed reductions in anxiety and depression and significant changes in weight status. That is, improvement in these psychological features was not directly related to weight normalization. This finding is consistent with conclusions from a recent systematic review (Kezelman et al., 2015) and seems to undermine assertions that experiences of anxiety for individuals with AN should be considered solely as concomitant effects of severe malnutrition and starvation that will naturally abate as a result of weight restoration. It is possible that the high levels of self-reported anxiety on admission reflected a temporary elevation related to the distressing context of being hospitalized for a psychiatric illness, an increase that naturally abated with time. Given the mounting literature espousing the frequent co-occurrence of anxiety and AN (Thornton et al., 2011; Frank and Kaye, 2012; Hildebrandt et al., 2012; Swinbourne et al., 2012), however, irrespective of treatment context, this explanation seems inadequate. Further research assessing the potential moderating factors resulting in this reduction is needed.

In addition, despite significant changes in global eating disorder symptomology and subscales relating to eating and restraint, no change in subscales relating to weight and shape concerns was evident. That is, while significant improvements in behavioral symptoms were observed, significant changes in cognitive factors underlying these eating disorder behaviors were not. Given that research increasingly asserts the inadequacy of behavioral change only in achieving long-term positive clinical outcomes (Fichter et al., 2006), this null finding may provide some insight into why targeting weight restoration as the primary objective is insufficient (Dancyger et al., 2013). Furthermore, there were no significant changes found in control over anxiety symptomology, as measured by the ACQ. While this may not be surprising given the prescriptive nature of this specific treatment setting focused on achieving medical stability, it may be important to consider the ways in which this may interact with achieving long-term clinical changes. Recent developments in clinical guidelines have suggested that once medical stability has been achieved there is no evidence to support hospitalization as the most appropriate treatment setting (Hay et al., 2014). Thus, while unquestionably nutritional rehabilitation is an essential component of effective treatment for AN, it needs to be embedded within a larger therapeutic framework drawing upon other evidence-based treatments that target specific psychological needs (e.g., long-standing anxiety disorders).

Interestingly, the adjunctive use of anti-psychotic medication during hospitalization did not interact with the observed reductions in anxiety. Given that anti-psychotic medications were prescribed to two-thirds of participants included in the current study and that a predominant reason espoused for prescribing anti-psychotic medications within this population is the expected reduction in anxiety and agitation (McKnight and Park, 2010), this null finding warrants further consideration. On the one hand, the administration of the medication to this population is in line with the limited evidence supporting the tolerability of olanzapine in underweight adolescents with AN (Gowers et al., 2010), patients tended not to be discharged from hospital on anti-psychotic medication. Thus, if the antipsychotic medications were not found to effectively reduce individuals’ experience of anxiety and were not formulated as part of a long-term treatment, findings from the current study may challenge the increasingly routine administration of antipsychotic medications within this population. Future research should more comprehensively assess the adjunctive use of these intervention across the course of treatment for AN.

A number of important contributions to understanding an individual’s experience during acute nutritional rehabilitation of AN have been raised in this study. Nevertheless, a number of limitations must be considered. This research was conducted in a specialist adolescent medical eating disorder unit that employs a very high caloric prescription, within revised clinical guidelines (The Society for Adolescent Health Medicine [SAHM], 2015), with the primary objective to provide rapid refeeding in a safe and effective manner. The specificity of this treatment setting may not be representative, thereby limiting the generalizability of current findings. It is also conceivable that the prescription of anti-psychotic medications resulted in an underestimation of the severity of anxiety symptomology. Further, while the sample size was within norms for research of AN populations, there may have been insufficient power to detect an effect. Finally, the scope of the current study was limited to an assessment of psychological features during hospitalization only. Taking power considerations, the lack of significant findings and the fact that two-thirds of patients were prescribed olanzapine, these data need to be regarded as tentative and in need of replication. Future studies may also want to address the relationship between caloric intake and anxiety rather than weight gain alone, as this may prove to be a more clinically significant marker of anxiety during treatment. Further research providing an assessment of anxiety experiences prior to admission, post-hospitalization, and over an extended follow-up period will also be important to inform a more thorough understanding of the longitudinal interaction of these features.

Notwithstanding these limitations, the current study is strengthened by the use of a prospective design, which allowed for a consideration of the psychological features associated with employing rapid refeeding protocol. The exclusive focus on an adolescent population was also a significant strength as, despite increased rates of incidence within this age range, prevalence data indicating peak rates of illness onset for AN during mid-adolescence, and suggestions that younger patients may be at greater risk from the acute effects of malnutrition and dehydration (Winston et al., 2012), there has been a paucity of evidence exclusively focused on this population.

The current study’s findings have important implications for the direction of future research. Most poignantly, there is an urgent need for randomized controlled trials that concurrently assess the progression of physiological and psychological treatment outcomes, alongside assessments regarding the optimal regime for nutritional rehabilitation. It is recommended that such research also incorporate an extended duration of follow-up to facilitate ongoing assessment of effective outcomes overtime. Given increasing suggestions that providing an exclusive focus on physiological rehabilitation before implementing any psychological interventions is unsupported (Strober and Johnson, 2012; Yager et al., 2012). Extending research in this manner may serve to delineate clinically meaningful outcomes within an inpatient setting and further contribute to the development of effective interventions.

## Ethics Statement

This study was carried out in accordance with the recommendation of the Western Sydney Local Health District Human Research Ethics Committee and the University of Sydney Human Research Ethics Committee. The protocol was approved by the Western Sydney Local Health District Human Research Ethics Committee and the University of Sydney Human Research Ethics Committee.

## Author Contributions

SK, PR, and ST contributed to research design and study methodology. SK, GA, and SC supported data collection. RC conducted statistical analyses. SK, RC, PR, CH, and ST contributed to study analysis and interpretation. All authors contributed to final manuscript revision.

## Conflict of Interest Statement

The authors declare that the research was conducted in the absence of any commercial or financial relationships that could be construed as a potential conflict of interest.
